# Targeting Lineage-Specific Transcription Factors and Cytokines of the Th17/Treg Axis by Novel 1,3,4-Oxadiazole Derivatives of Pyrrolo[3,4-*d*]pyridazinone Attenuates TNBS-Induced Experimental Colitis

**DOI:** 10.3390/ijms23179897

**Published:** 2022-08-31

**Authors:** Marta Szandruk-Bender, Benita Wiatrak, Stanisław Dzimira, Anna Merwid-Ląd, Łukasz Szczukowski, Piotr Świątek, Adam Szeląg

**Affiliations:** 1 Department of Pharmacology, Wroclaw Medical University, Mikulicza-Radeckiego 2, 50-345 Wrocław, Poland; 2 Department of Pathology, Wroclaw University of Environmental and Life Sciences, Norwida 31, 50-375 Wrocław, Poland; 3 Department of Medicinal Chemistry, Wroclaw Medical University, Borowska 211, 50-556 Wrocław, Poland

**Keywords:** experimental colitis, trinitrobenzenesulfonic acid, inflammatory bowel disease, pyrrolo[3,4-*d*]pyridazinone, inflammatory mediators, Th17/Treg axis, RORγt, Foxp3, IL-6, IL-23

## Abstract

The pharmacotherapy of inflammatory bowel disease (IBD) is still not fully effective and safe. Attempts to search for new IBD drugs remain an incessant research aim. One of the novel approaches is targeting the developmental pathway molecules and effector cytokines of Th17/Treg axis. This study aimed to elucidate the impact of new pyrrolo[3,4-*d*]pyridazinone derivatives, compounds **7b**, **10b**, or **13b**, on the course of experimental colitis in rats and to assess whether these new compounds may influence Th17/Treg axis. Rats were pretreated with studied compounds intragastrically before intrarectal administration of 2,4,6-trinitrobenzenesulfonic acid used for colitis induction. Body weight loss, disease activity index, colon index, and colon tissue damage were analyzed to evaluate the severity of colitis. The colonic levels of RORγt, STAT3, CCR6, Foxp3, IL-6, IL-10, IL-17, TNF-α, IL-23, and PGE_2_ were assessed. Pretreatment with compounds **7b** and **13b** alleviated the severity of colitis and concomitantly counteracted the increased levels of RORγt, STAT3, CCR6, IL-6, IL-17, IL-23, TNF-α, and PGE_2_. The beneficial effect of compounds **7b** and **13b** may be due to the decrease in the levels of Th17-specific transcription factors and cytokines. The studied compounds might therefore constitute a promising therapeutic strategy in Th17/Treg imbalance-driven inflammatory conditions such as IBD.

## 1. Introduction

Inflammatory bowel disease (IBD) refers to chronic and relapsing inflammatory disorders of the gastrointestinal tract [1]. Among the two main entities of IBD, Crohn’s disease (CD) is a transmural inflammation that may affect any part of the gastrointestinal tract, whereas ulcerative colitis (UC) is an inflammation of the colonic mucosa and submucosa [1,2]. Both forms of IBD can adversely impact all aspects of patients’ life, including a substantial deterioration in their quality of life [3,4,5]. While the incidence and prevalence of IBD remain highest in Western countries, they have recently increased in newly industrialized regions [6]. In the 21st century, inflammatory bowel disease has become a global disease, with over six million cases worldwide [7].

The dysregulated immune response is an indisputable factor relevant to the pathogenesis of IBD in which a disturbed balance between T helper 17 (Th17) and regulatory T (Treg) cells is considered crucial [3,8]. Th17 cells, secreting proinflammatory cytokines such as IL-17, IL-21, and TNF-α, represent pro-inflammatory cells, which, when in excess, contribute to induction and propagation of inflammation, and tissue damage, while Treg cells, secreting anti-inflammatory cytokines, e.g., IL-10, are essential for maintaining immune tolerance and restraining excessive Th17 cells responses. Thus, the Th17/Treg balance is crucial to intestinal homeostasis [3,8,9]. In IBD patients, the Th17/Treg balance is lost, and the inflamed intestinal mucosa tissues are characterized by a massive infiltration of Th17 cells and increased levels of Th17-related cytokines compared to that of healthy people [3,10].

Treatment of IBD has changed and improved over the years; however, it is still an incurable disorder [11]. The currently recommended treatment may cause serious and potentially irreversible side effects, especially during long-term administration. Moreover, there are still patients who do not respond to treatment or lose response over time [12,13]. With the above in mind, attempts to search for new, more efficient, and safer drugs for IBD are still valid and justified and remain an unmet clinical need. Given the crucial role ascribed to Th17/Treg cells imbalance in the development and maintenance of mucosal inflammation, targeting the developmental pathway molecules and effector cytokines of the Th17/Treg axis seems a promising strategy for preventing and treating IBD.

The differentiation of Th17 cells from naïve CD4^+^ T cells is directed by their master transcription factor retinoic acid-related orphan receptor γt (RORγt) and signal transducer and activator of transcription 3 (STAT3), while Treg cells are differentiated by forkhead box protein 3 (Foxp3). Moreover, IL-6 is crucial to the Th17 lineage development, whereas IL-23 is crucial to the maintenance and function of this subset [8,9,10,14]. In addition to the factors mentioned above, cyclooxygenase-2 (COX-2) and prostaglandins (PG), especially PGE_2_, also promote the development and function of Th17 cells [15,16]. Therefore, a new therapeutic approach in IBD may be based on inhibiting Th17- and enhancing Treg-specific transcription factors, as well as targeting Th17-related cytokines, cyclooxygenase, and its eicosanoid products [15,17].

In our previous studies, we have reported that the novel pyrrolo[3,4-*d*]pyridazinone derivatives strongly inhibit cyclooxygenase activity [18,19]. We have also shown that these novel pyrrolo[3,4-*d*]pyridazinone derivatives can alleviate inflammatory response in the carrageenan-evoked inflammation model and their mechanism of action might be related to the decrease in the PGE_2_, TNF-α, and MPO levels and the reduction in inflammatory cell infiltration in inflamed tissues [20]. Several other studies have indicated that compounds based on both pyridazinone [21] and 1,3,4-oxadiazole [22,23,24] scaffolds are able to interfere with the STAT3 pathway, which is involved in Th17 cells differentiation [8].

Based on these premises, we decided to assess whether these novel derivatives are able to alleviate inflammatory response by influencing the Th17/Treg axis. We chose these compounds, which turned out to be the most effective in our previous studies [18,19,20,25], i.e., 6-butyl-3,5,7-trimethyl-1-[[3-[(4-phenylpiperazin-1-yl)methyl]-2-thioxo-1,3,4-oxadiazol-5-yl]methoxy]pyrrolo[3,4-*d*]pyridazin-4-one and 6-butyl-3,5,7-trimethyl-1-[[3-[[4-(4-nitrophenyl)piperazin-1-yl]methyl]-2-thioxo-1,3,4-oxadiazol-2-yl]methoxy]pyrrolo[3,4-*d*]pyridazin-4-one and 6-butyl-1-[[3-[[4-(4-chlorophenyl)-4-hydroxy-1-piperidyl]methyl]-2-thioxo-1,3,4-oxadiazol-5-yl]methoxy]-3,5,7-trimethyl-pyrrolo[3,4-*d*]pyridazin-4-one (hereafter referred to as the compounds **7b**, **10b**, and **13b**, respectively).

The current study was undertaken to elucidate the effect of pretreatment with the new pyrrolo[3,4-*d*]pyridazinone derivatives, compounds **7b**, **10b**, or **13b**, on the course of intestinal inflammation in 2,4,6-trinitrobenzenesulfonic acid (TNBS) experimental colitis in rats, and to assess whether these new compounds may influence the Th17/Treg axis via targeting the Th17-related cytokines or inhibiting the Th17- and enhancing Treg-specific transcription factors.

## 2. Results

### 2.1. The Effects of Pyrrolo[3,4-d]pyridazinone Derivatives on Body Weight, Disease Activity Index, and Colon Index in Rats with TNBS-Induced Colitis

To assess the severity of colitis, the body weight loss, DAI, and colon index were analyzed, the results of which are presented in Table 1. The body weight of all rats increased from day 1 to 15, and there were no significant differences between the groups (*p* = NS). Conversely, significant body weight loss, DAI score increase, and colon index increase were noticed in TNBS-subjected rats compared with those in the control group (*p* < 0.001 in all comparisons), suggesting that TNBS effectively induced colitis and caused marked colonic injury. Following pretreatment with compound **7b** at both doses tested (10 or 20 mg/kg), the body weight loss was remarkably improved, and the DAI score and colon index were decreased compared to the TNBS group (*p* < 0.001, *p* < 0.01, *p* < 0.001 for the 10 mg/kg dose, respectively, and *p* < 0.001 for the 20 mg/kg dose in all cases). The difference from the control group was insignificant in all these cases (*p* = NS) except for the DAI value in the **7b**-10 group (*p* < 0.01). Either at a low or high dose, compound **10b** did not exert a significant effect on any TNBS-induced symptoms of colitis (*p* = NS vs. TNBS group and *p* < 0.01 vs. control group in all cases). Pretreatment with compound **13b** at a dose of 20 mg/kg, but not at a dose of 10 mg/kg, ameliorated the body weight loss (*p* < 0.05 vs. TNBS and control group), decreased the DAI score (*p* < 0.01 vs. TNBS and control group), and decreased the colon index (*p* < 0.001 vs. TNBS group, *p* = NS vs. control group). Compound **7b** at a low dose improved the body weight loss and colon index more effectively than compound **10b** in the corresponding dose (*p* < 0.01 in both cases). In turn, at a high dose, the effect of compound **7b** was greater than compound **10b** in the corresponding dose in the body weight loss, DAI, and colon index (*p* < 0.001 in all cases), and it was greater than compound **13b** in the corresponding dose in the body weight loss (*p* < 0.01) and DAI (*p* < 0.05).

### 2.2. The Effects of Pyrrolo[3,4-d]pyridazinone Derivatives on TNBS-Induced Macro- and Microscopic Colon Tissue Damage in Rats

**Figure 1:**
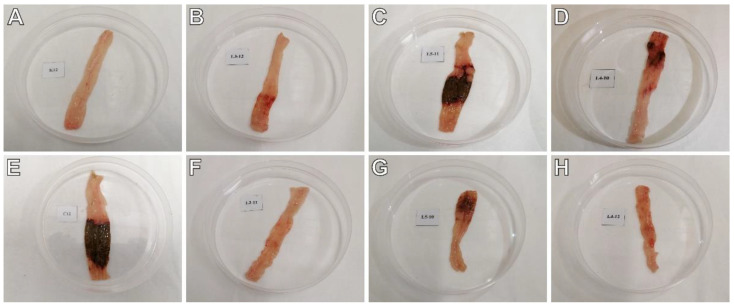
The gross appearance of the colon specimens revealed that compound **7b** (10 and 20 mg/kg) and compound **13b** (20 mg/kg) reduced macroscopically visible mucosal damage induced by TNBS. Control group (**A**); group receiving only TNBS (**E**); group receiving 10 mg/kg compound **7b** and TNBS (**B**); group receiving 20 mg/kg compound **7b** and TNBS (**F**); group receiving 10 mg/kg compound **10b** and TNBS (**C**); group receiving 20 mg/kg compound **10b** and TNBS (**G**); group receiving 10 mg/kg compound **13b** and TNBS (**D**); group receiving 20 mg/kg compound **13b** and TNBS (**H**).

**Figure 2:**
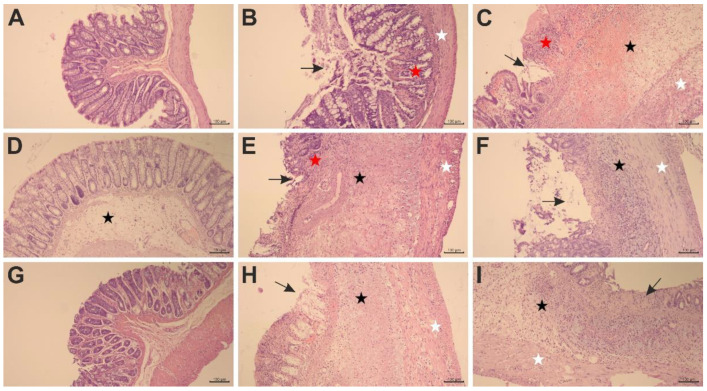
The photomicrographs of the colon tissues after hematoxylin–eosin staining revealed that compound **7b** (10 and 20 mg/kg) and compound **13b** (20 mg/kg) diminished TNBS-induced histological alterations; arrow in black, ulceration of the mucosa; star in red, inflammatory infiltrates within the lamina propria of the mucosa; star in black, edema within the submucosa; star in white, inflammatory infiltrates within the muscularis mucosa. Control group (**A**); group receiving only TNBS (**B**,**C**); group receiving 10 mg/kg compound **7b** and TNBS (**D**); group receiving 20 mg/kg compound **7b** and TNBS (**G**); group receiving 10 mg/kg compound **10b** and TNBS (**E**); group receiving 20 mg/kg compound **10b** and TNBS (**H**); group receiving 10 mg/kg compound **13b** and TNBS (**F**); group receiving 20 mg/kg compound **13b** and TNBS (**I**); magnification 100×.

To test whether the studied compounds counteract TNBS-induced lesions in the colon tissues, macroscopic and microscopic evaluations were performed. The scoring of colon tissue samples is shown in Table 2. Macroscopic changes in the colon involving bowel wall thickening, edema, hyperemia, and ulceration, were apparent in TNBS-subjected rats compared to the control rats (*p* < 0.001), in which no macroscopic lesions were observed (Figure 1A,E). Similarly, histological analysis revealed significant colon tissue injuries in the TNBS group contrasted to the control group (*p* < 0.001), in which the typical features of a normal colon with intact mucosa were observed (Figure 2A–C). From the H&E stained specimens, the colon tissues of the TNBS group exhibited disrupted colonic architecture, mucosal hyperemia, extensive ulceration involving mainly the epithelial layer (but also, in some studied specimens, all intestinal layers), massive mucosal to submucosal (and in the most severe cases transmural) infiltration of inflammatory cells, pronounced mucosal and submucosal edema with strong stimulation of the lymphatic apparatus, the loss of goblet cells, structural distortion of crypts, as well as desquamated areas or loss of the epithelium (Figure 2B,C). When compound **7b** was administered at a dose of 10 or 20 mg/kg prior to colitis induction, macro- and microscopic visible damage of the colon tissues was lessened compared to the group receiving only TNBS (*p* < 0.01 and *p* < 0.001 for the 10 mg/kg dose, respectively, and *p* < 0.001 for the 20 mg/kg dose in both cases). Rats in these two pretreated groups showed pronounced recovery of the colon tissues, with decreased ulceration extent and intensity, reduced edema and inflammatory cells infiltration, an increased amount of goblet cells, and restored epithelial cell layer (Figure 1B,F and Figure 2D,G). After pretreatment with compound **7b** at a high dose, the damaging effect of TNBS was almost completely abolished (Figure 1F and Figure 2G), and the histological features observed were similar to those of healthy control rats (*p* = NS). Similarly, compound **13b** at a dose of 20 mg/kg, but not at a dose of 10 mg/kg, diminished TNBS-induced macroscopic and microscopic colon lesions (*p* < 0.01 in both cases), demonstrating marked colon tissue recovery with decreased ulceration size and intensity, reduced edema and cellular infiltration but without or very slightly affecting the goblet cells (Figure 1D,H and Figure 2F,I). Either at a low or high dose, compound **10b** did not exert a significant effect on TNBS-induced macroscopic and microscopic colon damage (*p* = NS; Figure 1C,G and Figure 2E,H). At both doses tested, compound **7b** diminished TNBS-induced histological alterations more effectively than compound **10b** in the corresponding doses (*p* < 0.001 in both cases). Additionally, at a high dose, the effect of compound **7b** was greater than compound **13b** in the corresponding dose (*p* < 0.001).

### 2.3. The Effects of Pyrrolo[3,4-d]pyridazinone Derivatives on the Colonic Expression of RORγt, STAT3, CCR6, and Foxp3 in Rats with TNBS-Induced Colitis

**Figure 3:**
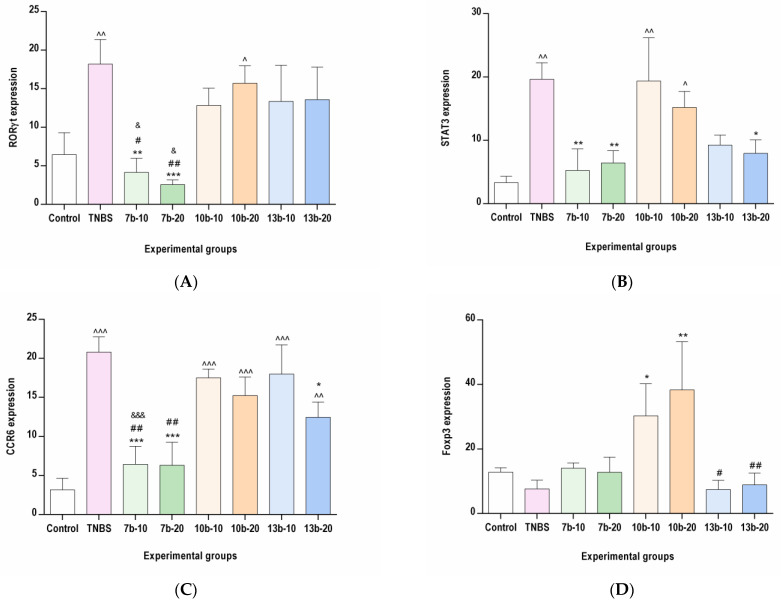
The impact of compounds **7b**, **10b** and **13b** on RORγt (**A**), STAT3 (**B**), CCR6 (**C**), and Foxp3 (**D**) expression in the colon tissues in experimental groups. Control, control group; TNBS, group receiving only TNBS; **7b**-10 and **7b**-20, groups receiving, respectively, 10 or 20 mg/kg of compound **7b** and TNBS; **10b**-10 and **10b**-20, groups receiving, respectively, 10 or 20 mg/kg of compound **10b** and TNBS; **13b**-10 and **13b**-20, groups receiving, respectively, 10 or 20 mg/kg of compound **13b** and TNBS. Data are presented as mean values ± SD; *n* = 10 for each group. Analyses were performed using the one-way ANOVA and Tukey’s post hoc test. Differences ^ *p* < 0.05 ^^ *p* < 0.01, ^^^ *p* < 0.001 vs. control group; * *p* < 0.05, ** *p* < 0.01, *** *p* < 0.001 vs. TNBS group; # *p* < 0.05, ## *p* < 0.01 vs. compound **10b** at the corresponding dose group; & *p* < 0.05, &&& *p* < 0.001 vs. compound **13b** at the corresponding dose group were deemed statistically significant.

**Figure 4:**
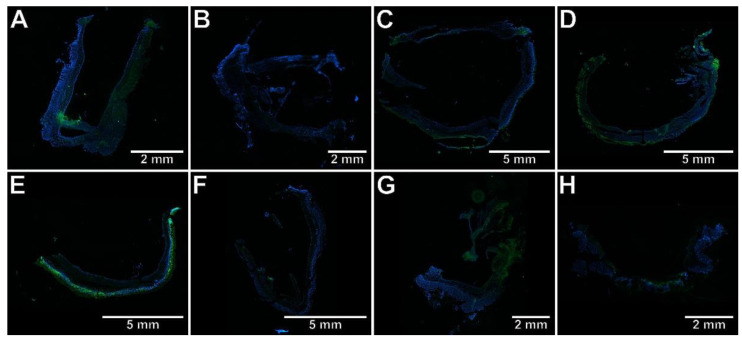
The photomicrographs of cryosections of the whole colon tissue samples after immunostaining with antibodies specific to RORγt fluorescently detected demonstrated that only compound **7b** counteracted TNBS-induced increase in RORγt expression. Control group (**A**); group receiving only TNBS (**E**); group receiving 10 mg/kg compound **7b** and TNBS (**B**); group receiving 20 mg/kg compound **7b** and TNBS (**F**); group receiving 10 mg/kg compound **10b** and TNBS (**C**); group receiving 20 mg/kg compound **10b** and TNBS (**G**); group receiving 10 mg/kg compound **13b** and TNBS (**D**); group receiving 20 mg/kg compound **13b** and TNBS (**H**); magnification 4×.

**Figure 5:**
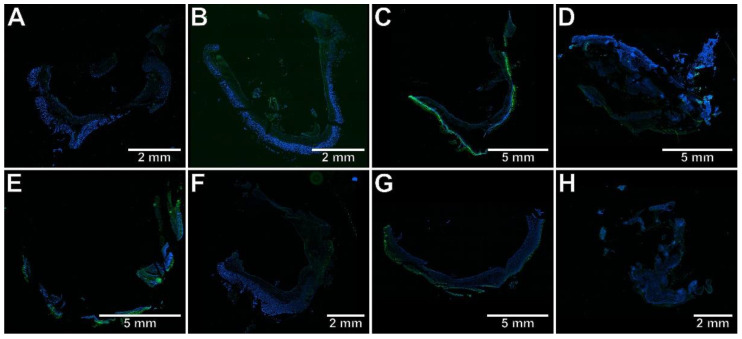
The photomicrographs of cryosections of the whole colon tissue samples after immunostaining with antibodies specific to STAT3 fluorescently detected demonstrated that compounds **7b** and **13b** counteracted TNBS-induced increase in STAT3 expression. Control group (**A**); group receiving only TNBS (**E**); group receiving 10 mg/kg compound **7b** and TNBS (**B**); group receiving 20 mg/kg compound **7b** and TNBS (**F**); group receiving 10 mg/kg compound **10b** and TNBS (**C**); group receiving 20 mg/kg compound **10b** and TNBS (**G**); group receiving 10 mg/kg compound **13b** and TNBS (**D**); group receiving 20 mg/kg compound **13b** and TNBS (**H**); magnification 4×.

**Figure 6:**
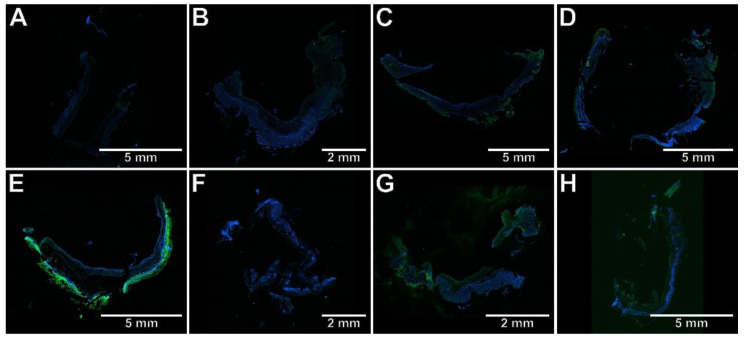
The photomicrographs of cryosections of the whole colon tissue samples after immunostaining with antibodies specific to CCR6 fluorescently detected demonstrated that compounds **7b** and **13b** counteracted TNBS-induced increase in CCR6 expression. Control group (**A**); group receiving only TNBS (**E**); group receiving 10 mg/kg compound **7b** and TNBS (**B**); group receiving 20 mg/kg compound **7b** and TNBS (**F**); group receiving 10 mg/kg compound **10b** and TNBS (**C**); group receiving 20 mg/kg compound **10b** and TNBS (**G**); group receiving 10 mg/kg compound **13b** and TNBS (**D**); group receiving 20 mg/kg compound **13b** and TNBS (**H**); magnification 4×.

**Figure 7:**
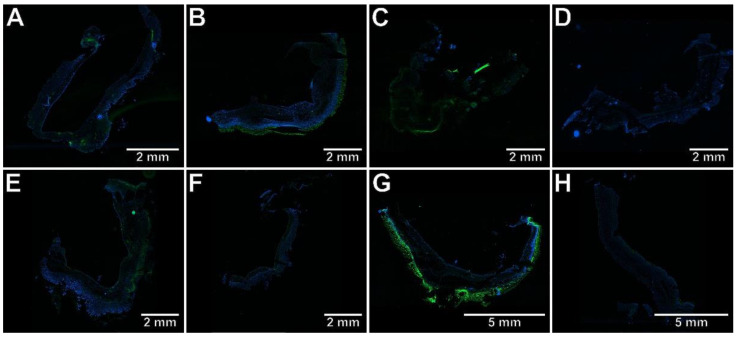
The photomicrographs of cryosections of the whole colon tissue samples after immunostaining with antibodies specific to Foxp3 fluorescently detected demonstrated that only compound **10b** counteracted TNBS-induced decrease in Foxp3 expression. Control group (**A**); group receiving only TNBS (**E**); group receiving 10 mg/kg compound **7b** and TNBS (**B**); group receiving 20 mg/kg compound **7b** and TNBS (**F**); group receiving 10 mg/kg compound **10b** and TNBS (**C**); group receiving 20 mg/kg compound **10b** and TNBS (**G**); group receiving 10 mg/kg compound **13b** and TNBS (**D**); group receiving 20 mg/kg compound **13b** and TNBS (**H**); magnification 4×.

To determine whether the protective effect of the studied compounds against TNBS-induced colitis is associated with the inhibition of Th17-specific or enhancement of Treg-specific transcription factors, the immunohistochemical analysis was performed, the results of which are presented in Figure 3, Figure 4, Figure 5, Figure 6 and Figure 7. The immunohistochemical evaluation showed that induction of colitis increased the expression of RORγt, STAT3, and CCR6 in the colon tissues compared to the control group (*p* < 0.01, *p* < 0.01, *p* < 0.001, respectively). At the same time, the colonic expression of Foxp3 was decreased, but not significantly (*p* = NS). At both examined doses, compound **7b** prevented the overexpression of RORγt, STAT3, and CCR6 in the colon tissues in comparison to the TNBS group (*p* < 0.01, *p* < 0.01, *p* < 0.001 for the 10 mg/kg dose and *p* < 0.001, *p* < 0.01, *p* < 0.001 for the 20 mg/kg dose, respectively). The difference to the control group was insignificant in all these cases (*p* = NS). Compound **10b** at a dose of 10 or 20 mg/kg increased Foxp3 expression compared to the TNBS group (*p* < 0.05 for the 10 mg/kg dose and *p* < 0.01 for the 20 mg/kg dose, respectively). Foxp3 expression in these two treated groups was not different from those in the control group (*p* = NS in both comparisons). Pretreatment with compound **13b** at a dose of 20 mg/kg only, prevented the TNBS-induced overexpression of STAT3 and CCR6 (*p* < 0.05 vs. TNBS group in both cases and *p* = NS, *p* < 0.01 vs. control group, respectively). At both doses tested, compound **7b** counteracted the increased RORγt expression more effectively than compounds **10b** and **13b** in the corresponding doses (*p* < 0.05 for the 10 mg/kg dose in both comparisons and *p* < 0.01, *p* < 0.05 for the 20 mg/kg dose, respectively). The effect of compound **7b** at a low dose on CCR6 expression was greater than compounds **10b** and **13b** in the corresponding doses (*p* < 0.01 and *p* < 0.001, respectively), and at a high dose, it was greater than compound **10b** in the corresponding dose (*p* < 0.01). In contrast, at both doses tested, the effect of compound **10b** on Foxp3 expression was greater than compound **13b** in the corresponding doses (*p* < 0.05 and *p* < 0.01, respectively).

### 2.4. The Effects of Pyrrolo[3,4-d]pyridazinone Derivatives on the Colonic Levels of IL-6, IL-17, IL-23, TNF-α, PGE_2_, and IL-10 in Rats with TNBS-Induced Colitis

**Figure 8:**
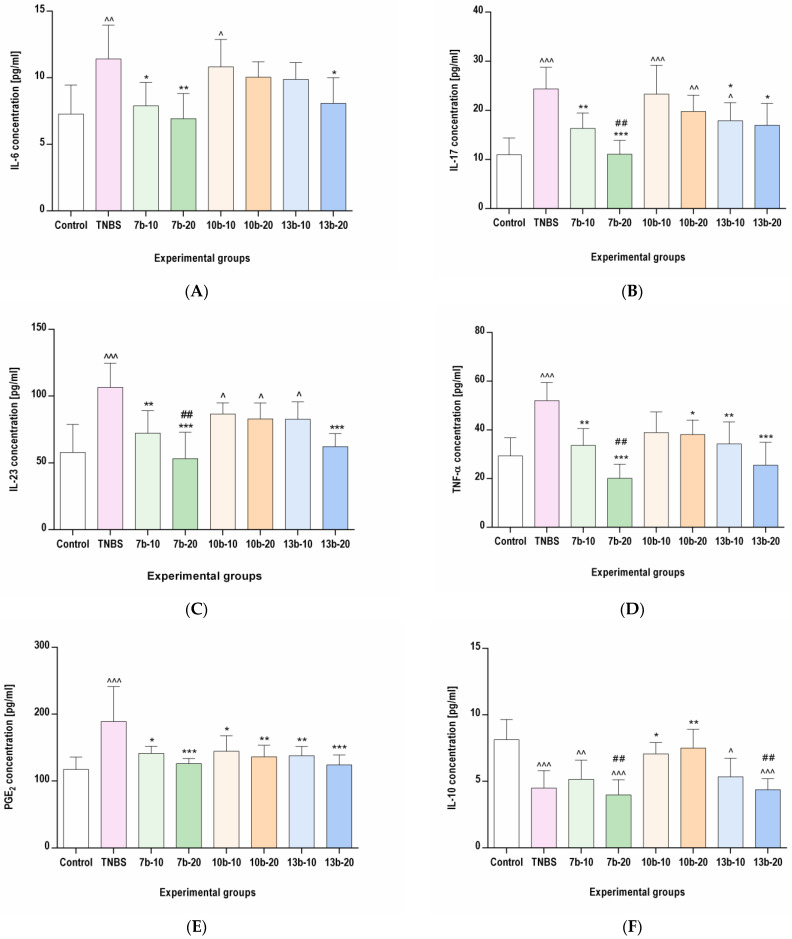
The impact of compounds **7b**, **10b,** and **13b** on IL-6 (**A**), IL-17 (**B**), IL-23 (**C**), TNF-*α* (**D**), PGE_2_ (**E**), and IL-10 (**F**) concentrations in the colon tissues in experimental groups. Control, control group; TNBS, group receiving only TNBS; **7b**-10 and **7b**-20, groups receiving, respectively, 10 or 20 mg/kg of compound **7b** and TNBS; **10b**-10 and **10b**-20, groups receiving, respectively, 10 or 20 mg/kg of compound **10b** and TNBS; **13b**-10 and **13b**-20, groups receiving, respectively, 10 or 20 mg/kg of compound **13b** and TNBS. Data are presented as mean values ± SD; *n* = 10 for each group. Analyses were performed using the one-way ANOVA and Tukey’s post hoc test. Differences ^ *p* < 0.05, ^^ *p* < 0.01, ^^^ *p* < 0.001 vs. control group; * *p* < 0.05, ** *p* < 0.01, *** *p* < 0.001 vs. TNBS group; ## *p* < 0.01 vs. compound **10b** at the corresponding dose group were deemed statistically significant.

To assess the influence of the studied compounds on inflammatory mediators specific to the Th17/Treg axis, ELISA and MILLIPLEX MAP assays were performed, the results of which are presented in Figure 8. The concentrations of proinflammatory mediators (IL-6, IL-17, IL-23, TNF-α, and PGE_2_) were significantly increased by TNBS administration when compared to the control group (*p* < 0.01, *p* < 0.001, *p* < 0.001, *p* < 0.001, and *p* < 0.001, respectively). In contrast, the level of anti-inflammatory IL-10 was markedly decreased by TNBS administration compared to the control group (*p* < 0.001). Pretreatment with compound **7b** at both doses tested (10 or 20 mg/kg) prevented the increase in all studied proinflammatory mediators and normalized their concentrations in the colon tissues compared to the TNBS group (*p* < 0.05, *p* < 0.01, *p* < 0.01, *p* < 0.01, *p* < 0.05 for the 10 mg/kg dose and *p* < 0.01, *p* < 0.001, *p* < 0.001, *p* < 0.001, *p* < 0.001 for the 20 mg/kg dose, respectively). All studied proinflammatory mediators concentrations in these two pretreated groups were not different from the control group (*p* = NS in all cases). Compound **7b** did not affect the concentration of IL-10 at either low or high doses (*p* = NS vs. TNBS group and *p* < 0.01, *p* < 0.001 vs. control group, respectively). Compound **10b**, at a high dose (20 mg/kg) given before TNBS administration, prevented the increase in TNF-α and PGE_2_ and, concomitantly, prevented the decrease in IL-10 colonic tissue levels in comparison to the colitis group (*p* < 0.05, *p* < 0.01, *p* < 0.01 vs. TNBS group, respectively, and *p* = NS vs. control group in all cases). Compound **10b**, at a low dose (10 mg/kg), protected only from the PGE_2_ increase and IL-10 decrease (*p* < 0.05 vs. TNBS group and *p* = NS vs. control group in both cases). Pretreatment with compound **13b** at a high dose counteracted the increased levels of all studied proinflammatory markers concentrations in the colon tissues compared to the group receiving only TNBS (*p* < 0.05, *p* < 0.05, *p* < 0.001, *p* < 0.001, *p* < 0.001, respectively, and *p* = NS vs. control group in all cases), while the administration of compound **13b** at a low dose counteracted only the increased IL-17, TNF-α, and PGE_2_ levels (*p* < 0.05, *p* < 0.01, *p* < 0.01 vs. TNBS group and *p* < 0.05, *p* = NS, *p* = NS vs. control group, respectively). Compound **13b**, at either low or high doses, did not affect IL-10 level (*p* = NS vs. TNBS group and *p* < 0.05, *p* < 0.001 vs. control group, respectively). At a high dose, compound **7b** counteracted the increased IL-17, IL-23, and TNF-α levels more effectively than compound **10b** in the corresponding dose (*p* < 0.01 in all cases). In turn, at a high dose, the effect of compound **10b** on IL-10 was greater than that of compounds **7b** and **13b** in the corresponding doses.

### 2.5. Multi-Criteria Decision Analysis (MCDA)

**Figure 9:**
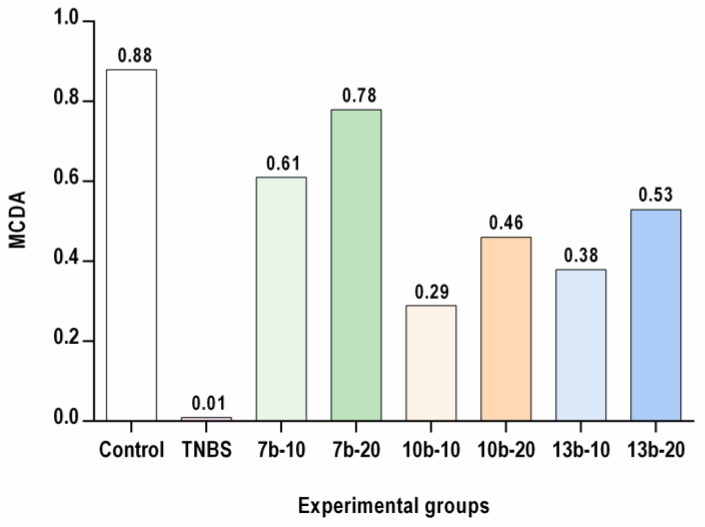
Multi-criteria decision analysis (MCDA) comparing the effect of the application of the studied compounds on the decrease/increase in the measured parameters compared to the control (group of rats without the studied compounds and without induction of colitis with TNBS solution). The TNBS group: rats with induced colitis without the studied compounds compared to the control.

The results achieved from each assay (body weight loss, DAI, colon index, macro- and microscopic assessments of colon tissues, RORγt, STAT3, CCR6, and Foxp3 expression, IL-6, IL-17, IL-23, TNF-α, PGE_2_, and IL-10 levels) for studied pyrrolo[3,4-*d*]pyridazinone derivatives were analyzed using MCDA to compare their pharmacological activity. The MCDA results (Figure 9) demonstrated that the greatest protective effect against the experimental colitis was found for compound **7b** at a dose of 20 mg/kg, followed by compound **7b** at a dose of 10 mg/kg and compound **13b** at the dose of 20 mg/kg. At both doses tested, compounds **7b** and **13b** exerted a greater effect than compound **10b**. Additionally, compound **7b** had a greater effect than compound **13b.**

## 3. Discussion

Animal models of IBD are a valuable implement for evaluating new therapeutic strategies for IBD and analyzing the possible mechanism of action of a given drug, even though none of the individual models reflects all aspects, stages, and complexity of the human disease [26]. Among the various experimental models of IBD, the TNBS-induced colitis model is widely used as it shares many biochemical and immunological characteristics and symptoms with human disease, especially human CD [27,28]. In this model, colonic inflammation is induced by intrarectal administration of ethanol TNBS solution. After the interruption of the colonic mucosal epithelial barrier by ethanol, TNBS reaches the lamina propria and haptenizes colonic or colonic microbiota proteins with trinitrophenyl moiety to render them immunogenic, thereby triggering an immune response reflected by a transmural colonic inflammation with dense infiltration of CD4^+^ T cells, neutrophils, macrophages, and the secretion of proinflammatory mediators [27,29].

Following administration of TNBS solution, as happened in this study, rats develop acute colitis hallmarks, including body weight loss, inconsistent stool formation, diarrhea, and rectal bleeding [29] used in various scales assessing the severity of inflammatory bowel disease, e.g., DAI score [30]. The results found in this study revealed that compound **7b** given at a dose of 10 or 20 mg/kg and compound **13b** at a dose of 20 mg/kg alleviated the course of experimental colitis, as evidenced by improved body weight loss as well as by the reduction in DAI, colon index, and macroscopically visible lesions. These beneficial effects have been confirmed by histological analysis, which showed that after pretreatment with compound **7b** (10 or 20 mg/kg) and compound **13b** (20 mg/kg), the grade and extent of intestinal inflammation were reduced, as manifested by a marked decrease in inflammatory cells infiltration, ulceration, and edema. Moreover, histopathology of the colon tissues showed that compound **7b** at a high dose almost completely reversed the TNBS-induced alterations, thereby restoring nearly normal colon tissue architecture. This suggests, along with the DAI score, which in the **7b**-20 group was close to that of the healthy control group, that compound **7b** at a high dose not only limited but also prevented experimental colitis. The achieved results are in line with our earlier findings that pretreatment with the new pyrrolo[3,4-*d*]pyridazinone derivatives prevented the increase in inflammatory cell influx into the inflamed tissues and morphological alterations in the carrageenan-induced inflammation [20]. As far as we know, this study is the first to elucidate the effect of pyrrolo[3,4-*d*]pyridazinone derivatives on experimental colitis in rats, revealing that pretreatment with these new compounds can ameliorate clinical and histological symptoms induced by TNBS.

It has been recently shown that among many innate and adaptive inflammatory cells infiltrating intestinal tissues, both in IBD and experimental TNBS-induced colitis, the IL-17-producing Th17 cells are of particular importance due to their proinflammatory role in the mucosal immune response [31,32]. The intestinal mucosal immune system must maintain concomitantly a state of tolerance towards intestinal antigens and the ability to protect the host against pathogens. This balance is reached by several mechanisms, including reciprocal regulation of proinflammatory Th17 and suppressive Treg cell lineages [33]. In IBD, this balance is lost with a shift towards the proinflammatory Th17 side. It leads to an overproduction of proinflammatory cytokines from constantly accumulating Th17 cells with resultant inflammation, which far exceeds the immune tolerance of Treg, thereby forming a vicious cycle promoting mucosal inflammation [8,9].

**Figure 10:**
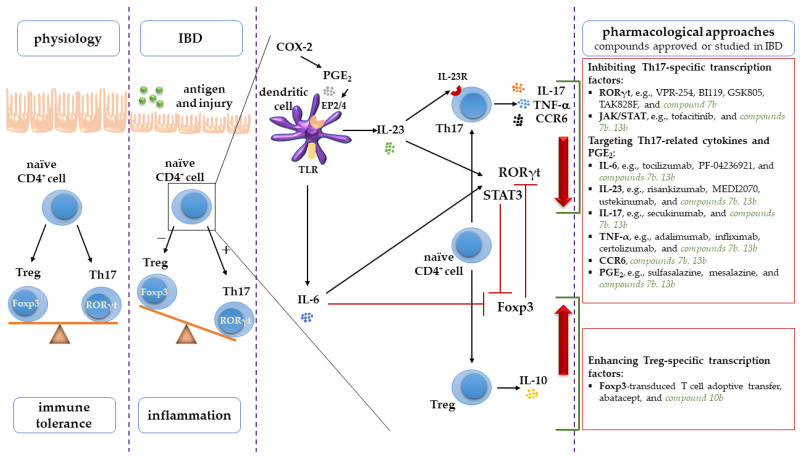
Th17/Treg axis, essential for intestinal immune homeostasis when balanced, crucial in IBD pathogenesis when dysregulated, reciprocally interconnected regarding Th17 and Treg cells development, and new pharmacological target in IBD [3,12,13,16,17,39,43,44,45,46,47,48,49,50,51,52,60,65]. Abbreviations: COX-2, cyclooxygenase-2; CCR6, C-C chemokine receptor 6; EP2/4, prostaglandin E receptor 2 and 4; Foxp3, forkhead box protein 3; IBD, inflammatory bowel disease; IL, interleukin; IL-23R, IL-23 receptor; PGE_2_, prostaglandin E_2;_ ROR, retinoic acid-related orphan receptor γt; STAT3, signal transducer and activator of transcription 3, Th17, T helper 17 cell, Treg, regulatory T cell; TLR, Toll-like receptor; TNF-α, tumor necrosis factor α.

Although Th17 and Treg cells fulfill opposed roles in inflammation, they are both developed from naïve CD4^+^ T cells. Their differentiation is reciprocally interconnected, requires lineage-specific transcription factors, and depends on the surrounding microenvironment (mainly the presence of proinflammatory mediators) [8]. Th17 cells differentiation is directed by their master transcription factor retinoic acid-related orphan receptor γt (RORγt), and signal transducer and activator of transcription 3 (STAT3) after the exposure to proinflammatory cytokines, especially IL-6 and IL-23 [14,33]. IL-6 is a crucial signaling protein that promotes Th17 polarization via inducing in naïve CD4^+^ T cells RORγt and STAT3 expression [14,32], which in turn bind to the promoter region of the *Il17* gene and mediate IL-17 transcription [34]. Unlike IL-6, IL-23 is not strictly required for Th17 differentiation because of the lack of IL-23 receptor (IL-23R) in naïve T cells (Figure 10). However, in newly differentiating Th17 cells, IL-6 and RORγt induce the expression of IL-23R to make them receptive to signals delivered by IL-23 [8,33]. IL-23 signaling is then critically needed for the maintenance, expansion, and pathogenicity of the Th17 cell population by further enhancing RORγt and STAT3 expression [14,33].

Altogether, IL-6, IL-23, RORγt, and STAT3 constitute a complex interconnected network of the Th17 cell lineage regulators, whose altered expression may be related to a persistent Th17 cell response [34]. Indeed, the expression of these Th17 developmental pathway-specific molecules is highly up-regulated in the inflamed intestinal tissues and correlated with disease activity in IBD patients and experimental colitis [35,36,37,38,39,40,41]. Thus, normalization of the increased levels of IL-6, IL-23, RORγt, and STAT3 may be crucial to inhibit the differentiation of Th17 cells and, given their prominent role in IBD pathogenesis, may prevent or at least alleviate the symptoms of intestinal inflammation. Recently, some novel biologics and small molecule drugs targeting the Th17 developmental pathway, have been developed and investigated in several preclinical and clinical studies [42]. For most of them, the results, while promising, are still preliminary. Monoclonal antibodies against IL-6, tocilizumab, and PF-04236921, as well as against the p19 subunit of IL-23, risankizumab, and MEDI2070, have shown potential efficacy in CD patients in the early stages of studies [43,44,45,46], while ustekinumab, binding the p40 subunit of IL-23, has already been approved for the treatment of CD patients [47]. In turn, the novel small molecule inhibitors of RORγt, VPR-254, BI119, GSK805, and TAK828F, were effective in murine models of IBD [48,49,50,51]. Another synthetic small molecule drug, tofacitinib, a Janus kinase (JAK) inhibitor that reduces STAT3 expression, is under investigation as a possible treatment for IBD [39,52]. In the current study, new synthesized small molecule compounds also targeted the Th17 developmental pathway. Pretreatment with compound **7b** at a dose of 10 or 20 mg/kg counteracted the increased IL-6 and IL-23 levels as well as RORγt and STAT3 expression, whereas compound **13b** at a dose of 20 mg/kg counteracted the increased IL-6, IL-23 levels and STAT3 expression in the colon tissues as compared to TNBS-subjected rats. As mentioned above, among the compounds tested, these were the ones that alleviated the course of experimental colitis. It is worth emphasizing that in this study, a decrease in RORγt expression was observed only in the case of pretreatment with compound **7b**, which is the most efficient among the studied compounds and alone not only limited but also prevented experimental colitis. This may be attributed to the fact that downregulating the RORγt expression affects not only Th17 differentiation and IL-17 production but also the expression of IL-23R, critically involved in Th17 pathogenicity, which is under the transcriptional control of RORγt [8]. It can be therefore assumed that targeting RORγt is crucial in alleviating symptoms of experimental colitis. To the best of our knowledge, our study is the first to evaluate the effect of 1,3,4-oxadiazole derivatives of pyrrolo[3,4-*d*]pyridazinone on the expression of Th17-specific transcription factors RORγt and STAT3 and the Th17 cell-polarizing cytokine IL-23 in an experimental colitis model, showing that pretreatment with these new compounds may prevent the increase in RORγt, STAT3, and IL-23 tissue levels. Thus far, it has been reported by other authors that compounds based on pyridazinone [21] or 1,3,4-oxadiazole [22,23,24] scaffolds act as STAT3 inhibitors in computational and in vitro studies only. Concerning IL-6, our results are in line with Tan et al. [53], whose findings show that other diversely substituted pyridazinone derivatives decreased IL-6 levels in experimental colitis induced by dextran sodium sulfate (DSS).

The essential role that the Th17 cell lineage plays in IBD depends on the expression of proinflammatory effector molecules, including downstream effector cytokines IL-17 and TNF-*α*, as well as C-C chemokine receptor 6 (CCR6), which all mediate tissue inflammatory infiltration and tissue damage [14,54,55]. Both IL-17 and TNF-α are pleiotropic cytokines that can cause or exacerbate intestinal inflammation by recruiting and activating neutrophils and other inflammatory cells and by triggering the release of other proinflammatory cytokines via activation of NF-κB and MAPK pathways [32]. Moreover, IL-17 and TNF-*α* can further induce RORγt and STAT3 expression, thereby forming a positive loop, amplifying the immune response, prolonging inflammation, and causing further local tissue damage [54]. CCR6, highly expressed on Th17 cells, drives leukocyte migration to inflamed tissues within the gut mucosa and plays an essential role in intestinal inflammation contributing to IBD pathogenesis [55]. IL-17, TNF-*α*, and CCR6 protein and mRNA levels are elevated and positively correlated with the severity of both IBD and experimental colitis [31,32,35,36,54,55,56,57]. For these reasons, IL-17, TNF-*α*, and CCR6 may serve as potential therapeutic targets to control gut inflammation and mitigate its course. The benefits of TNF-α regulation have already been proven by the use of anti-TNF-α drugs in the treatment of IBD patients [12,13]. In the present study, the alleviation of TNBS-induced symptoms and normalization of developmental pathway molecule levels by compound **7b** at a low or high dose and compound **13b** at a high dose were accompanied by a decrease in the tissue levels of IL-17, TNF-α, and CCR6. It may imply that targeting developmental pathway proteins may enable suppression of the production of several downstream effector proinflammatory molecules at the same time. The achieved results corroborated our previous findings that pyrrolo[3,4-*d*]pyridazinone derivatives can decrease TNF-*α* tissue levels in the inflamed tissues in various inflammation models in vivo [20,25] and those of Tan et al. [53], whose results show that some other derivatives of pyridazinone can decrease the TNF-*α* colonic level in DSS colitis model. To the best of our knowledge, our study is the first to evaluate the effect of pyrrolo[3,4-*d*]pyridazinone derivatives on IL-17 level and CCR6 expression, indicating that the studied compounds can lessen the levels of these Th17-specific effector molecules. Intriguingly, compound **13b** at a low dose decreased the levels of IL-17 and TNF-*α* but did not alleviate the symptoms of colitis. It may be explained by the fact that inhibition of upstream cytokines and transcription factors of the Th17 lineage is of crucial importance for symptom relief [58]. Moreover, as others have highlighted [9,14,59], IL-17 has a dual context-dependent nature. IL-17, produced in an IL-23-independent manner by *γδ* T cells, is gut protective and indispensable to the defense against pathogens. In turn, IL-17, produced in an IL-23-dependent manner by Th17, is gut pathogenic and promotes inflammation (so-called IL-23/Th17 axis). Hence, it can be concluded that after administration of compound **13b** at a low dose, the decrease in IL-17 level without affecting IL-23 concentration is not sufficient to decrease inflammation and does not reduce the development of colitis. Similarly, the IL-17 neutralizing agent, secukinumab, is ineffective in CD patients [60]. We believe that lowering IL-17 and IL-23 levels concomitantly, as with both **7b** at both doses and **13b** at a high dose, minimizes colon tissue inflammation, supposedly leaving the IL-23-independent IL-17 intact.

Notably, it has been recently shown that other non-cytokine inflammatory mediators, including PGE_2_, released in response to proinflammatory cytokines found within the gut microenvironment can exacerbate inflammation and disease severity in both IBD and experimental colitis by enhancing the Th17 response [61]. PGE_2_ increases the production of IL-6 and IL-23 in macrophages and dendritic cells and upregulates the expression of IL-23R on differentiating naïve T cells via the EP2 and EP4 receptors signaling pathway. Additionally, PGE_2_ synergizes and amplifies the action of IL-6 and IL-23, increasing the expression of RORγt and CCR6. This promotes the differentiation and expansion of Th17 cells and subsequently induces their effector cytokines production and release, including IL-23-dependent IL-17 [16,61,62]. Lee et al. [15] revealed that along with IL-23, PGE_2_ signaling is crucial to making Th17 cells pathogenic in immune inflammation in vivo. In the present study, all compounds tested prevented the TNBS-induced increase in PGE_2_ levels. This is in line with our recent reports showing that the new pyrrolo[3,4-*d*]pyridazinone derivatives at a dose of 10 or 20 mg/kg counteracted the increased levels of PGE_2_ in inflamed tissues in vivo [20,25]. However, only in the case of pretreatment with compound **7b** at a low or high dose or **13b** at a high dose, a decrease in PGE_2_ level was accompanied by a reduction in the course of inflammation. This can be explained by the fact that PGE_2_ itself does not promote Th17 differentiation; however, it significantly enhances IL-23-induced production of IL-17 [63,64].

As discussed above, Th17 cells are controlled and inhibited from overactivation by Treg cells [65,66]. The differentiation of Treg cells is governed by forkhead box protein 3 (Foxp3) transcription factor, which is also required for their immune suppressive function [67]. Foxp3 is able to bind to RORγt and prevent its transcriptional activity, thereby inhibiting Th17 and favoring Treg cells development as well as inhibiting RORγt-driven IL-17 production [68]. Since the decreased intestinal expression of Foxp3, which occurs in IBD and experimental colitis, can impair the intestinal immune tolerance and activate inflammation, it can be assumed that preventing the reduction in Foxp3 expression may alleviate intestinal inflammation [66,69]. The results obtained in the presented study show that at both doses tested, compound **10b**, but not **7b** or **13b**, administered before TNBS, counteracted the decrease in Foxp3 expression. Surprisingly, this was not accompanied by an improvement in clinical or histological symptoms in the course of experimental colitis. A possible explanation may be the phenomenon of Treg cell plasticity [10,33]. The differentiation of Treg cells is not static, they can re-differentiate into Th17 cells [33,70]. Yang et al. [67] reported that IL-6 overpasses the suppressive effect of Foxp3 on RORγt and, together with IL-23 [71], can reprogram fully differentiated Treg cells towards the Th17 lineage, both in murine models and human IBD. Quite possibly, in our study, IL-6 and IL-23, which levels were not decreased by compound **10b**, promote a transcriptional program in which Treg cells can be converted to Th17 cells. It is noteworthy that even though proinflammatory cytokines are the main driving force behind the plasticity of Treg cells, other environmental cues can tune the re-differentiation of these cells [72]. More recent evidence reveals that CCR6 expression is characteristic not only of Th17 cells but also of Treg, and CCR6 signaling is able to direct Treg cells towards the Th17 cells via enhancing the expression of RORγt in them [72,73]. In our study, apart from IL-6 and IL-23, CCR6 level was also not decreased by compound **10b**. It may also promote Treg re-differentiation into Th17 cells. Taken together, this may mean that Foxp3-mediated inhibition of RORγt prevails under non-inflammatory conditions, whereas RORγt and RORγt suppression of Foxp3 predominate in the specific inflammatory milieu present in the tissues in the state of IBD. Indeed, in IBD patients, Foxp3^+^IL-17-producing Treg cells are observed in the inflamed intestines tissues compared with their slightly or non-inflamed counterparts, which suggests the involvement of Treg plasticity and Foxp3^+^IL-17-producing Treg cells in intestinal inflammation pathogenesis [72,74].

A key Treg-derived factor that restrains Th17-mediated inflammation of the intestinal tissues is IL-10 able to reduce the expression of proinflammatory mediators and inhibit antigen presentation [75]. Impaired IL-10 signaling is involved in the pathogenesis of IBD [76], and mice deficient in IL-10 are known to develop spontaneous intestinal inflammation [75]. In this study, only compound **10b** prevented the TNBS-induced decrease in the level of IL-10. However, as with Foxp3, following pretreatment with this compound, there was no improvement in the symptoms of colitis. A possible explanation is that IL-10 alone fails to suppress all the proinflammatory mediators involved in intestinal inflammation and is insufficient to counter aberrant immune responses in IBD. Additionally, IL-10 release is a feedback mechanism in response to elevated levels of proinflammatory cytokines, particularly IL-6. Since IL-10 release acts as one of the mechanisms that provide effector cells with a mechanism to regulate their inflammatory activity, the intensity of inflammatory response correlates with IL-10 levels—the greater the inflammation, the greater IL-10 levels [77]. It may explain that in the current study, the elevated level of IL-10 was only observed after administration of compound **10b**, which did not normalize proinflammatory cytokines concentrations. As far as we know, this is the first study assessing the influence of pyrrolo[3,4-*d*]pyridazinone derivatives on Foxp3 and IL-10 expression in experimental colitis, which reveals that preventing the decrease in Foxp3 and IL-10 expression in a proinflammatory molecule environment is insufficient to alleviate the symptoms of inflammation and, presumably, would need to go hand-in-hand with inhibition of proinflammatory mediators [67,72,73].

## 4. Materials and Methods

### 4.1. Drugs and Chemicals

The studied compounds—novel 1,3,4-oxadiazole derivatives of pyrrolo[3,4-*d*]pyridazinone named **7b**, **10b**, and **13b**—were obtained from the Department of Medicinal Chemistry, Wroclaw Medical University, Poland. The design, synthesis, experimental data, and spectra of the studied compounds and the intermediates have already been described [18]. Their structure and purity were established and confirmed by different spectroscopic and analytical techniques. Every new reported compound was determined to have a purity of >95%, based on its NMR or ESI-MS spectra. The following chemicals were used in this study: 2,4,6-trinitrobenzenesulfonic acid, normal goat serum (NGS), and 4’,6-diamidino-2-phenylindole (DAPI) were purchased from Sigma Aldrich (Steinheim, Germany); isoflurane was supplied from CP-Pharma (Burgdorf, Germany); medetomidine hydrochloride was purchased from Orion Pharma (Warszawa, Poland); carboxymethylcellulose (CMC), formalin, ethanol, methanol, Tween 20, Triton X-100, and bovine serum albumin (BSA) were purchased from PolAura (Olsztyn, Poland); pentobarbital sodium was obtained from Biowet (Puławy, Poland); normal saline was supplied from Polpharma S.A. (Starogard Gdański, Poland); O.C.T. compound was purchased from Thermo Fisher Scientific (Waltham, MA, USA); anti-RORγt, anti-STAT3, anti-CCR6, Goat Anti-Rabbit IgG H&L, and Donkey Anti-Goat IgG H&L antibodies were purchased from Abcam (Cambridge, UK); anti-Foxp3 antibody was supplied from Novus Biologicals (Abingdon, UK); Vectashield was supplied from Vector Laboratories (Burlingame, CA, USA). Other chemicals used were included in the commercially available kits.

### 4.2. Animals

Wistar male rats (210–260 g, Animal Research Center at Wroclaw Medical University, Wrocław, Poland) were kept two per cage in polypropylene cages with enrichments in the standard laboratory conditions with a 12 h light/dark cycle, a humidity of 55–60% and a temperature of 21–24 °C, with access to water ad libitum and free access to standard pelleted animal feed (Agropol, Motycz, Poland), except for 12 h deprivation before TNBS administration. Rats were adapted to the laboratory condition for seven days before commencing the experiments.

### 4.3. Ethical Statement

The experimental protocol was approved (Resolution No. 005/2020 of 15 January 2020) by the Local Ethics Committee for Animal Experiments in Wrocław at Hirszfeld Institute of Immunology and Experimental Therapy of the Polish Academy of Sciences (Wrocław, Poland). The animal care and all experimental procedures were conducted according to the applicable international, national, and institutional guidelines, including the Act of 15 January 2015 on the protection of animals used for scientific and educational purposes (Journal of Laws of 2015, item 266) and the EU directive 2010/63/EU.

### 4.4. Experimental Design

**Figure 11:**
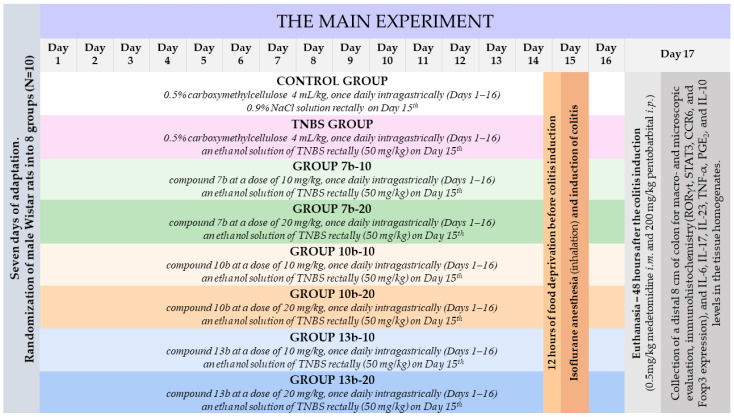
Flowchart of the experimental design for the present study.

As shown in Figure 11, after seven days of adaptation, rats were randomly assigned to eight groups (ten rats per group) organized as follows:

- One group pretreated with 0.5% CMC solution (vehicle) intragastrically (i.g.) and receiving once normal saline per rectum (p.r.), i.e., the control group;
- One group pretreated with 0.5% CMC i.g. and receiving once TNBS solution p.r., i.e., the colitis (TNBS) group;
- Six groups pretreated i.g. with compound **7b** or **10b** or **13b** at the doses of 10 or 20 mg/kg and receiving once TNBS solution p.r., i.e., the **7b**-10, **7b**-20, **10b**-10, **10b**-20, **13b**-10, **13b**-20 groups, respectively.

The doses of the studied substances were chosen based on previous studies [20,25]. To assess whether the studied compounds prevent the occurrence or reduce TNBS-induced colon damage, which most closely resembles the prevention of human IBD exacerbations after the remission period, a 16-day pretreatment with the studied compounds was chosen. The 0.5% CMC solution or studied compounds (suspended in 0.5% CMC) were administered once daily via a gastric tube (FST, Foster City, CA, USA) in a volume of 4 mL/kg for 16 consecutive days. Under inhalation isoflurane anesthesia, normal saline or TNBS solution was given rectally on the 15th day of the experiment, after 12 h of food deprivation. Animals were weighed and observed daily. Rats were sacrificed 48 h after induction of colitis by intramuscular injection of medetomidine (0.5 mg/kg) followed by intraperitoneal injection of pentobarbital (200 mg/kg). Then, the distal 8 cm of the colon of each rat was extracted, placed on an ice-cold plate, opened longitudinally, cleaned, rinsed with saline, weighed, and macroscopically analyzed. The colon samples were then divided into three parts for different analyzes. The first part was fixed in 4% buffered formalin, processed by routine techniques and stained with H&E, for histopathological examination. The second part was homogenized (Homogenizer PRO250, PRO Scientific Inc., Oxford, CT, USA), with the obtained supernatants stored at −80 °C for inflammatory markers evaluation. The third part was snap frozen, mounted in an O.C.T. embedding compound, and sectioned using a cryostat (Leica CM1850, Leica Biosystems, Nussloch, Germany) for immunohistochemistry studies.

### 4.5. Induction of Experimental Colitis

Experimental colitis was elicited employing the procedure originally described by Morris et al. [78]. Briefly, rats were anesthetized by isoflurane inhalation (4–5% for induction, 2–3% for maintenance). Subsequently, TNBS (50 mg/kg) dissolved in 50% ethanol (*v*/*v*) was instilled into the colon using a flexible catheter carefully inserted into the lumen of the colon through the rectum with the tip positioned approximately 8 cm proximal to the anus. For the next 5 min, the animals were in the Trendelenburg position to avoid leakage of the instilled solution.

### 4.6. Assessment of the Body Weight, Disease Activity Index, and Colon Index

The difference in the body weight between days 15 and 17 of the experiment was analyzed. In addition to body weight changes, stool consistency and blood in the stool were monitored. Based on these three parameters (which are analogous to the clinical presentation of human IBD), the disease activity index (DAI) was calculated as the sum of points ranging from 0 (unaffected) to 12 (severe colitis) scored by evaluation of each parameter following a previously reported scale [30]. The scoring scale of DAI parameters was as follows: weight loss (%): 0, 0–1%; 1, 1–4%; 2, 4–8%; 3, 8–12%; 4, >12%. Stool: 0, normal; 1, soft and shaped; 2, loose; 3, between; 4, diarrhea. Bloody stool: 0, none; 1, between; 2, slight; 3, between; 4, gross bleeding. The weight of each extracted 8 cm-long specimen of the colon was measured. The colon index was then calculated as the ratio of colon weight to total body weight on day 17 of the experiment.

### 4.7. Macro- and Microscopic Assessment of the Colon Tissues

The collected 8 cm colon sections were examined macroscopically immediately after resection according to the 0–5 scale described by Galvez et al. [79], which considers the area of inflammation and the degree of ulcerations. The scoring scale of the macroscopic evaluation was as follows: 0, no damage; 1, hyperemia, no ulcers; 2, linear ulcer with no significant inflammation; 3, linear ulcer with inflammation at one site; 4, two or more sites of ulceration or inflammation and ulceration or inflammation extending < 1 cm; 5, two or more major sites of ulceration or inflammation extending > 1 cm along the length of the colon. The colon sections were assessed in a blinded manner, each time under the same conditions, under the same artificial lighting, and at the same workplace. The photos were taken with a Nikon D3500 + AF-P DX 18–55 VR camera (Nikon, Warszawa, Poland) placed on a tripod, always at the same distance from the photographed object, under the same artificial lighting, at the same workplace.

Then, colon sections were collected for histological analysis. The material was fixed in 4% buffered formalin, embedded in paraffin, sectioned into 4 μm-thick slices (Sakura Accu-Cut SRM, Netherlands), and stained with hematoxylin–eosin (H&E) to assess colonic damage. The colon tissue sections were evaluated in a blinded manner by the experienced pathologist using standard light microscopy. For each colon tissue sample, 3 sections (extreme left section, middle, and extreme right section) were examined (extreme, i.e., approx. 3–4 mm from the edge of the tissue). All sections were examined in different fields of view, under different magnifications (50×, 100×, 200×) taking pictures of the most representative places at a magnification of 100×. Microphotographs of colon samples taken with a digital camera were assessed based on the image analysis system (Olympus BX53 microscope coupled to a UC90 camera with software Olympus cellSense Standard ver. 1.0 Olympus Soft Imaging Solutions GmbH, Germany). Histological damage was appraised using the scoring scale, which considers the extent of inflammation and ulceration, edema, and inflammatory cell infiltration, based on the criteria previously described by Arribas et al. [80]. The result was calculated as the sum of points ranging from 0 (unaffected) to 25 (severe colitis) scored by evaluation of each criterion. The criteria for assessing microscopic damage and the numerical rating score were as follows: in the mucosal epithelium and lamina propria: ulceration (0–4), mononuclear cell infiltration (0–3), polymorphonuclear cell infiltration (0–3); in submucosa: edema (0–3), mononuclear cell infiltration (0–3), polymorphonuclear cell infiltration (0–3); in muscular layer: mononuclear cell infiltration (0–3), polymorphonuclear cell infiltration (0–3). The scoring scale was: 0, none; 1, mild; 2, moderate; 3, severe; 4, full thickness.

### 4.8. Immunohistochemical Assessment of RORγt, STAT3, CCR6, and Foxp3 Expression in the Colon Tissues

**Figure 12:**
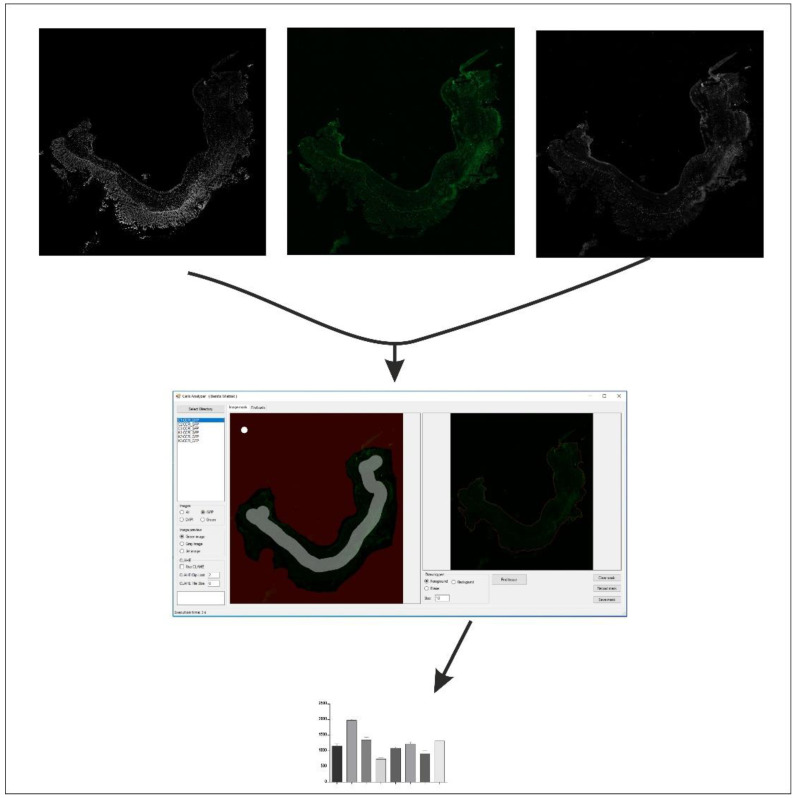
Scheme of the process of quantitative analysis of the level of protein expression performed with the use of proprietary software; magnification 4×.

Immunohistochemical detection of RORγt, STAT3, CCR6, and Foxp3 levels was performed in 4 μm cryosections of colonic tissue, in a blinded manner. The presence of the investigated markers was carried out in the freshly collected, snap-frozen material without fixation in formalin. This assay was conducted by immunostaining methods according to standard procedures. Endogenous tissues background control was performed in IHC studies as some tissues possess natural biological properties that emit natural fluorescence. Prior to IHC staining, the prepared tissues were examined microscopically using fluorescent illumination. On this basis, it was ensured that there was no signal inherent in the tissue itself. The sections cut on the cryostat were fixed with chilled to −20 °C 100% methyl alcohol for 5 min and then washed with 0.1% Tween 20 in PBS (PBST) three times for 5 min. Cell membrane permeabilization was performed with 0.1% Triton X-100 solution in PBS for 10 min at room temperature. The blocking of non-specific antibody binding was performed in a solution containing 1% BSA and 10% NGS in PBST for 30 min. Antibodies were diluted in the appropriate proportions in 1% BSA in PBST and incubated with colonic tissue sections overnight at room temperature in the dark. Anti-STAT3 antibody was dissolved to the final concentration of 5 µg/mL, and anti-CCR6 to the concentration of 0.5 µg/mL. In turn, anti-RORγt antibody was diluted at a ratio of 1:1000, and anti-Foxp3, 1:200. The next day, colonic tissue sections were washed 3 times for 5 min in PBS, and secondary antibodies were used (except for anti-Foxp3, which was already conjugated with Alexa Fluor 488), also prepared in 1% BSA, 10% NGS solution in PBST for 1 h, at room temperature, in the dark. As secondary antibodies, Goat Anti-Rabbit IgG H&L or Donkey Anti-Goat IgG H&L conjugated with Alexa Fluor 488 dye was used, depending on the host species of primary antibody. DAPI counterstaining was also performed to allow complete cell counts. After rinsing the colonic tissue sections, they were mounted in Vectashield mounting medium to prevent discoloration, allowing long-term storage of the stained specimens. Additionally, the no primary antibody control was performed, for all four parameters tested, the specificity of the immune labeling was checked by incubation with PBS instead of the specific primary antibody. For all four parameters, we analyzed the whole colon tissue section for ten samples in each group by using the EVOS^®^ FL Auto Imaging System (Thermo Fisher Scientific, Waltham, MA, USA) which allows you to scan a large area of a sample and obtain multiple images to build a tiled and stitched image. Quantitative analysis of the protein expression level was performed using proprietary software. The color intensity for individual cells was measured (detected by contrast staining), and only on this basis, the average intensities for the individual images were calculated. The photos taken were analyzed using this proprietary software using grayscale, DAPI, and GFP photos, and the so-called mask to determine the tissue area for analysis. After deciding the tissue area, the algorithm calculated the number of cells per % tissue (Figure 12).

### 4.9. Assessment of IL-6, IL-17, IL-23, TNF-α, PGE_2_, and IL-10 Levels in the Colon Tissues

Cytokines IL-6, IL-17, TNF-α, and IL-10 were determined in the obtained supernatants with the MILLIPLEX MAP Rat Cytokine/Chemokine Magnetic Bead Panel (Merck Millipore, Darmstadt, Germany) according to the manufacturer’s instructions. The analysis and quantification were performed by MAGPIX Instrument (Merck Millipore, Darmstadt, Germany) using BelysaTM Software version 1.0.19. The concentrations of IL-23 and PGE_2_ were measured with enzyme-linked immunosorbent assay (ELISA) kits: Rat IL-23 ELISA Kit, Rat PGE_2_ ELISA Kit (Cloud-Clone Corp., Katy, TX, USA) following the manufacturer’s instructions. All concentrations were expressed as pg/mL.

### 4.10. Statistical Analysis

The data obtained for all groups were checked for normal distribution and equality of variance with the Shapiro–Wilk and Brown–Forsythe tests, respectively. The *p* values for both tests for all groups were *p* > 0.05; therefore, statistical analyses were performed with parametric tests, and data are presented as mean values ± standard deviation (SD). The one-way analysis of variance (ANOVA) followed by Tukey’s multiple comparison test was applied to analyze the statistical significance of differences among studied groups.

To summarize the results obtained in all assays performed and to compare the pharmacological properties of the studied compounds, the multiple-criteria decision analysis (MCDA) based on the weighted sum model was carried out. In the MCDA analysis, the same weights were assumed for all tests. The results of the individual tests were additionally normalized prior to the analysis so that the maximum effect achieved in one test should have the same significance for the final result as the maximum result achieved in other tests.

Data analysis was performed using GraphPad Prism version 8.0 (GraphPad Software, San Diego, CA, USA) and Tibco Statistica version 13.3 (StatSoft, Kraków, Poland) with a *p*-value < 0.05 considered as the significance level.

## 5. Conclusions

The findings provided herein point to the conclusion that two of the studied novel pyrrolo[3,4-*d*]pyridazinone derivatives, compounds **7b** and **13b**, attenuate intestinal inflammation in the preclinical model of IBD. Their activity seems to be linked to the decrease in the levels of transcription factors and cytokines specific to the Th17 cell lineage. Further, it may be inferred that these compounds, by concomitantly targeting transcription factors and upstream cytokines of the Th17 cell lineage, represent a multi-factorial pharmacological approach different than targeting downstream effector Th17-related cytokines alone. The studied compounds might therefore constitute a promising therapeutic strategy in Th17/Treg imbalance-driven inflammatory conditions such as IBD, with the potential not only to relieve symptoms but also to modify the pathogenesis of the disease. However, additional investigations are needed to increase knowledge about the mechanisms involved in the beneficial effect shown by the studied compounds.

## Figures and Tables

**Table 1:** The impact of compounds **7b**, **10b**, and **13b** on the body weight loss, DAI, and colon index in experimental groups.

Parameter	Group	Value	*p*-Value (vs. Control)	*p*-Value (vs. TNBS)	*p*-Value (vs. Comp. 10b-10)	*p*-Value (vs. Comp. 10b-20)	*p*-Value (vs. Comp. 13b-10)	*p*-Value (vs. Comp. 13b-20)
Body weight loss (g)	Control	20.50 ± 2.25	-	<0.0001	<0.0001	0.0097	0.0001	0.0187
	TNBS	−10.78 ± 1.39	<0.0001	-	NS	NS	NS	0.0355
	**7b**-10	10.80 ± 3.55	NS	0.0006	0.0047	NS	NS	NS
	**7b**-20	25.00 ± 4.64	NS	<0.0001	<0.0001	0.0003	<0.0001	0.0025
	**10b**-10	−7.78 ± 3.96	<0.0001	NS	-	NS	NS	NS
	**10b**-20	2.00 ± 1.36	0.0097	NS	NS	-	NS	NS
	**13b**-10	−3.60 ± 2.36	0.0001	NS	NS	NS	-	NS
	**13b**-20	5.00 ± 1.91	0.0187	0.0355	NS	NS	NS	-
DAI	Control	0.00 ± 0.00	-	<0.0001	<0.0001	<0.0001	<0.0001	0.0011
	TNBS	5.70 ± 1.25	<0.0001	-	NS	NS	NS	0.0040
	**7b**-10	3.40 ± 1.35	0.0040	0.0011	NS	NS	NS	NS
	**7b**-20	1.65 ± 0.82	NS	<0.0001	0.0001	0.0005	0.0005	0.0390
	**10b**-10	4.60 ± 1.35	<0.0001	NS	-	NS	NS	NS
	**10b**-20	4.10 ± 1.29	<0.0001	NS	NS	-	NS	NS
	**13b**-10	4.10 ± 1.60	<0.0001	NS	NS	NS	-	NS
	**13b**-20	3.60 ± 1.27	0.0011	0.0040	NS	NS	NS	-
Colon index	Control	0.002 ± 0.000	-	<0.0001	<0.0001	0.0004	0.0009	NS
	TNBS	0.007 ± 0.002	<0.0001	-	NS	NS	NS	0.0001
	**7b**-10	0.003 ± 0.001	NS	<0.0001	0.0005	0.0211	NS	NS
	**7b**-20	0.002 ± 0.000	NS	<0.0001	<0.0001	0.0002	0.0004	NS
	**10b**-10	0.007 ± 0.002	<0.0001	NS	-	NS	NS	NS
	**10b**-20	0.005 ± 0.001	0.0004	NS	NS	-	NS	NS
	**13b**-10	0.005 ± 0.002	0.0009	NS	NS	NS	-	NS
	**13b**-20	0.004 ± 0.001	NS	0.0001	NS	NS	NS	-

Notes: Control, control group; TNBS, group receiving only TNBS; **7b**-10 and **7b**-20, groups receiving, respectively, 10 or 20 mg/kg of compound **7b** and TNBS; **10b**-10 and **10b**-20, groups receiving, respectively, 10 or 20 mg/kg of compound **10b** and TNBS; **13b**-10 and **13b**-20, groups receiving, respectively, 10 or 20 mg/kg of compound **13b** and TNBS. Data are presented as mean values ± SD; *n* = 10 for each group. Analyses were performed using the one-way ANOVA and Tukey’s post hoc test; *p*-value < 0.05 deemed as the significance level; NS, not significant.

**Table 2:** The impact of compounds **7b**, **10b**, and **13b** on macroscopic damage of the colon tissues and microscopic damage of the colon tissues in H&E staining in experimental groups.

Parameter	Group	Value	*p*-Value (vs. Control)	*p*-Value (vs. TNBS)	*p*-Value (vs. Comp. 10b-10)	*p*-Value (vs. Comp. 10b-20)	*p*-Value (vs. Comp. 13b-10)	*p*-Value (vs. Comp. 13b-20)
Macroscopic damage score (0–5 points)	Control	0.00 ± 0.00	-	<0.0001	<0.0001	<0.0001	<0.0001	0.0009
	TNBS	3.50 ± 1.08	<0.0001	-	NS	NS	NS	0.0016
	**7b**-10	1.93 ± 0.84	0.0003	0.0048	NS	NS	NS	NS
	**7b**-20	1.36 ± 0.69	NS	<0.0001	NS	NS	NS	NS
	**10b**-10	3.00 ± 0.82	<0.0001	NS	-	NS	NS	NS
	**10b**-20	2.36 ± 0.75	<0.0001	NS	NS	-	NS	NS
	**13b**-10	2.71 ± 0.70	<0.0001	NS	NS	NS	-	NS
	**13b**-20	1.79 ± 0.49	0.0009	0.0016	NS	NS	NS	-
Microscopic damage score, H&E staining (0–25 points)	Control	0.00 ± 0.00	-	<0.0001	<0.0001	<0.0001	<0.0001	<0.0001
	TNBS	15.78 ± 4.27	<0.0001	-	NS	NS	NS	0.0018
	**7b**-10	9.89 ± 3.33	<0.0001	0.0001	0.0004	NS	NS	NS
	**7b**-20	2.89 ± 1.27	NS	<0.0001	<0.0001	<0.0001	<0.0001	<0.0001
	**10b**-10	15.44 ± 2.35	<0.0001	NS	-	NS	NS	0.0045
	**10b**-20	12.11 ± 1.76	<0.0001	NS	NS	-	NS	NS
	**13b**-10	12.11 ± 2.93	<0.0001	NS	NS	NS	-	NS
	**13b**-20	10.78 ± 1.39	<0.0001	0.0018	0.0045	NS	NS	-

Notes: Control, control group; TNBS, group receiving only TNBS; **7b**-10 and **7b**-20, groups receiving, respectively, 10 or 20 mg/kg of compound **7b** and TNBS; **10b**-10 and **10b**-20, groups receiving, respectively, 10 or 20 mg/kg of compound **10b** and TNBS; **13b**-10 and **13b**-20, groups receiving, respectively, 10 or 20 mg/kg of compound **13b** and TNBS. Data are presented as mean values ± SD; *n* = 10 for each group. Analyses were performed using the one-way ANOVA and Tukey’s post hoc test; *p*-value < 0.05 deemed as the significance level; NS, not significant.

## Data Availability

The data underlying this article will be shared upon request to the corresponding author.
